# Aquaporin-3 in Cancer

**DOI:** 10.3390/ijms18102106

**Published:** 2017-10-07

**Authors:** Saw Marlar, Helene H. Jensen, Frédéric H. Login, Lene N. Nejsum

**Affiliations:** Department of Clinical Medicine, Bartholins Allé 6, Aarhus University, 8000 Aarhus C, Denmark; sawmarlar@gmail.com (S.M.); helenehalkjaer@clin.au.dk (H.H.J.); frederic.login@clin.au.dk (F.H.L.)

**Keywords:** aquaporin-3, cancer cell migration, metastasis, AQP3

## Abstract

Increasing evidence suggests that the water/glycerol channel aquaporin-3 (AQP3) plays a pivotal role in cancer metastasis. AQP3 knockout mice were resistant to skin tumor formation and overexpression correlated with metastasis and poor prognosis in patients with breast or gastric cancer. In cultured cancer cells, increased AQP3 expression stimulated several intracellular signaling pathways and resulted in increased cell proliferation, migration, and invasion as well as aggravation of epithelial-to-mesenchymal transition. Besides AQP facilitated water transport at the leading edge of migrating cells, AQP3 signaling mechanisms are beginning to be unraveled. Here, we give a thorough review of current knowledge regarding AQP3 expression in cancer and how AQP3 contributes to cancer progression via signaling that modulates cellular mechanisms. This review article will expand our understanding of the known pathophysiological findings regarding AQP3 in cancer.

## 1. Introduction

**Figure 1:**
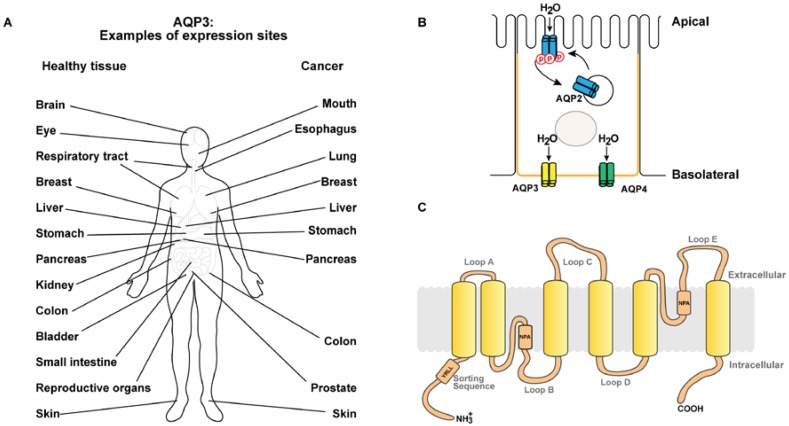
Aquaporin-3 expression sites, subcellular localization, and topology. (**A**) Schematic showing examples of aquaporin-3 (AQP3) expression sites as well as examples of cancers, where increased expression of AQP3 has been reported. (**B**) Schematic of a kidney collecting duct principal cell. Transepithelial water transport is facilitated by apical AQP2 allowing entry of water into the collecting duct principal cell and AQP3 and AQP4 in the basolateral plasma membrane domain (orange), which facilitate exit of water. AQP2 plasma membrane localization is regulated by phosphorylation (denoted by p in the schematic) mediating shuttling between intracellular vesicles and the apical plasma membrane. (**C**) Schematic showing the topology of AQP3. Similar to other AQPs, AQP3 has six transmembrane domains and intracellular COOH and NH_3_^+^ termini. Two membrane-integrated helices with the conserved Asn-Pro-Ala (NPA) motifs generate the pore. AQP3 contains a basolateral sorting motif (YRLL) in the NH_3_^+^-terminal.

Water accounts for approximately 50–60% of body mass, and regulation of water homeostasis is vital for all living organisms (for review [1,2]). Aquaporins (AQPs) are water channels that facilitate transepithelial water transport across plasma membranes following an osmotic gradient [3]. AQPs are glycosylated integral membrane proteins and widely expressed in bacteria (for review [4]), yeast [5], plants [6] (for review [7]), and mammals (for review [1,2,8]). Thirteen mammalian AQP isoforms have been identified (AQP0–12) (for review [2,9]) and are expressed in various tissues and organs including kidneys (for review [1,2]), eyes [10], brain [11,12] (for review [13,14]), lungs [15], skin [16,17], liver [18], sweat glands [16], and secretory glands [19] (see Figure 1A,B). AQPs are homotetramers and each monomer consists of six transmembrane domains with the N- and C-termini protruding into the cytoplasm. Highly conserved Asn-Pro-Ala (NPA) motifs in loops B and E bend into the lipid bilayer forming a water-selective pore, hence each monomer contains a pore permeable to water [20,21] (see Figure 1C for a schematic 2D topology of AQP3). According to their permeability characteristics, AQPs are grouped into aquaporins (AQP1, AQP2, AQP4, AQP5, AQP6, and AQP8, permeable to water) and aquaglyceroporins (AQP3, AQP7, AQP9, and AQP10, permeable to water, glycerol, urea [22], and other solutes). AQP11 and AQP12 are intracellular AQPs where the physiological roles are less well characterized [23] (for review [24,25]). Besides water, some AQPs have been reported to facilitate transport of H_2_O_2_ (AQP3 [26,27] and AQP8 [28]), ions (AQP0 [29,30], AQP1 [31,32,33] and AQP6 [34]) (for review [35]), and gas (AQP1 [36,37,38]).

Although homologous, AQPs have distinct subcellular localization (Figure 1B). Polarized localization to specific plasma membrane domains enables epithelial-specific functions. For example, AQP1 is both apically and basolaterally localized in renal proximal tubule cells [39,40]. AQP3 and AQP4 are localized to basolateral membranes in renal collecting duct principal cells [41,42], however, AQP4 is predominantly basal (for review [43]). AQP2 is localized to the apical membrane and subapical vesicles in collecting duct principal cells [44] (Figure 1B). AQP5 is both apical and basolateral in the last portion of the excretory duct and exclusively apical in the first portion [16]. Moreover, AQP6 is localized to intracellular vesicles in renal collecting duct intercalated cells [45]. Short-term regulation of AQP2 levels in the apical plasma membrane of renal collecting duct principal cells occurs via shuttling of vesicles upon vasopressin stimulation, which increases intracellular cyclic adenosine monophosphate (cAMP) [46]. Moreover, short-term cAMP stimulation regulates plasma membrane diffusion of AQP2 [47,48], AQP3 [49] and AQP5 [50]. Long-term vasopressin stimulation regulates both mRNA and protein levels of AQP2 [51] and AQP3 [41]. Moreover, although destined for the same plasma membrane domain, AQP3 and AQP4 are differentially sorted in the secretory pathway [52] and also localize differently in the lateral and basal membranes [53] (for review [43]).

### AQP3 Expression Pattern

AQP3 is expressed in basolateral plasma membranes of multiple human epithelia (see Figure 1A for examples of expression sites). In the gastrointestinal tract, AQP3 is expressed in gastric mucosal tissue [17,54], in ileum [17,54] and in distal colon [17,54,55] where it contributes to water and glycerol transport in the gastrointestinal tract (for review [56,57]). In addition, AQP3 is expressed in the upper and lower airways [58] where it facilitates osmotic water transport across the airway epithelial cells (for review [59]). Furthermore, AQP3 is expressed in brain [54,60], breast [54], liver [61], pancreas [54,62], ovary [54], prostate [63], bladder [64], and many more epithelia [54]. In the kidney, AQP3 is expressed in the basolateral plasma membrane of collecting duct principal cells, where it is involved in fine-tuning of urine concentration [22,65,66,67] (see Figure 1B for schematic of a collecting duct principal cell). AQP3 is also strongly expressed in the plasma membrane of keratinocytes in the basal and spinous layers of epidermis [68,69] (for review [70]). Here, AQP3 serves as a water- and glycerol-transporter [71,72] facilitating skin hydration [73] and may also be involved in cell migration during wound healing [74]. AQP3 is also expressed in corneal epithelia where studies of AQP3 knock-out mice showed that AQP3 facilitates water and glycerol transport as well as participates in wound healing following injury [75].

## 2. AQP3 in Cancer

Recently, implication of several aquaporins in cancer has been reported (for review [76,77,78]) and increasing evidence strongly suggests that AQP3 plays a pivotal role in cancer progression and metastasis (for review [76,77,78,79,80,81]). A study of AQP3 knockout mice revealed that AQP3 was necessary for skin tumor development, since AQP3 knockout mice did not develop skin tumors after exposure to a tumor initiator [82]. Moreover, overexpression and ectopic expression of AQP3 has been observed in several cancers (see Figure 1A for examples of expression sites), where it contributes to metastasis, proliferation, and epithelial-to-mesenchymal transition (EMT) [83,84].

### 2.1. Expression Pattern in Cancer Tissue

AQP3 was found to be overexpressed in lung cancer [85,86], colon cancer [87], esophageal and oral squamous cell carcinoma [88]. Moreover, in hepatocellular carcinoma [89] and pancreatic ductal adenocarcinoma [90], AQP3 was dually overexpressed with AQP5. Overexpression of AQP3 was reported in 65 out of 89 patients diagnosed with gastric adenocarcinoma, and this overexpression correlated with histological classification, lymph node metastasis, and lymphovascular invasion leading to shorter patient survival compared to those with lower AQP3 expression [84]. Similarly, in a study of patients with breast invasive ductal carcinoma, increased expression of AQP3 was observed. This was associated with higher histological grade and increased spreading to lymph nodes highlighting the importance of AQP3 in breast cancer progression [83]. In a study of 94 patients with bladder carcinoma, high tumor stage was associated with decreased levels of AQP3 expression [91]. It is not known why AQP3 seems to be decreased in bladder cancer, but is overexpressed in multiple other cancers.

Together, these reports suggest AQP3 as a player in cancer. Why and how the expression of AQP3 is altered in multiple cancers is not clear. Epidermal growth factor (EGF) and estrogen both contribute to cancer development and have been suggested as upstream regulators of AQP3 expression. In cultured colorectal cancer [87], ovarian cancer [92], and pancreatic cancer cells [93], EGF treatment increased AQP3 expression. In breast cancer cells expressing estrogen receptor, stimulation with estrogen transcriptionally upregulated the levels of AQP3 [83].

It is also not clear how overexpressed or ectopically expressed AQP3 accelerates cancer progression. As discussed below, AQP3 expression affected cellular functions commonly associated with cancer progression, including proliferation, motility, and EMT. In addition, some potential downstream targets have been identified [94,95].

### 2.2. Cancer Cell Proliferation

Cancer cells are characterized by accelerated cellular proliferation. Observations from cell cultures suggest that AQP3 promotes cancer cell proliferation (for review [96]). AQP3 overexpression enhanced the proliferation of gastric cancer cells SGC7901 and MGC803 while silencing of endogenous AQP3 decreased proliferation [84]. Likewise, high expression of AQP3 increased the proliferation rate of PC12 rat pheochromocytoma cells [97] (for review [96]). Moreover, when AQP3 protein levels were decreased by knockdown, gastric cancer SGC7901 and MGC803 cells formed fewer spheroids, indicating that AQP3 promotes spheroid formation of these cells [98] and AQP3 knockdown in pancreatic BXPC3 and HPAFII cancer cells, decreased cell proliferation [99].

### 2.3. Cancer Cell Migration

**Figure 2:**
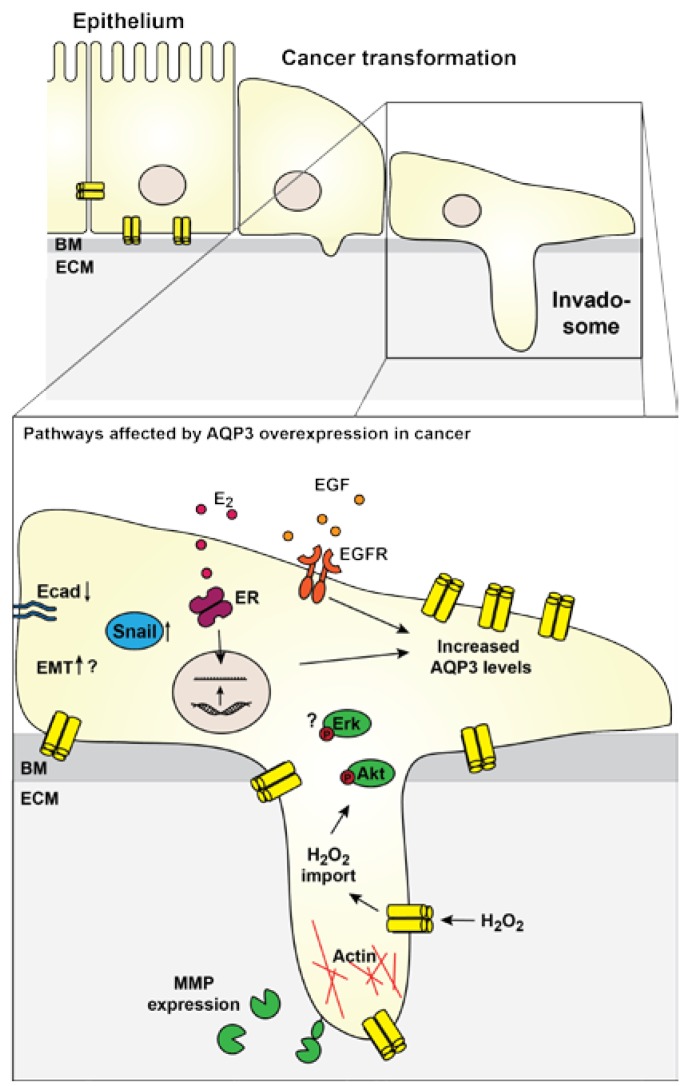
Schematic showing signaling affecting AQP3 overexpression and the downstream effectors. In cancer, AQP3 is often upregulated. Upregulation may be stimulated by EGF signaling. Moreover, in ER-positive breast cancer, estrogen signaling increased AQP3 transcription. Some downstream effects of AQP3 in cancer have been identified or suggested. AQP3-mediated H_2_O_2_ uptake was necessary for increased protein kinase B (Akt) phosphorylation, and AQP3 may also affect extracellular signal-regulated kinase (Erk)1/2 phosphorylation through so far unknown mechanisms. AQP3 overexpression was necessary for increased expression of MMPs, which promote cancer cell invasiveness. Actin is polymerized in the leading edge of migrating cells, and AQP3 localizes here as well. AQP3 overexpression facilitated downregulation of E-cadherin and up-regulation of Snail suggesting a contribution of AQP3 to EMT. BM: Basement membrane; E_2_: Estradiol; Ecad: E-cadherin; ECM: Extra-cellular matrix; EGF: Epidermal growth factor; EGFR: Epidermal growth factor receptor; EMT: Epithelial-to-mesenchymal transition; ER: Estrogen receptor; MMP: Matrix-metallo protease.

Increased motility of cancer cells is an important aspect of cancer development. Motile cancer cells may migrate away from the primary tumor and establish metastases, which significantly complicates treatment strategies and lowers survival. Studies of other AQPs have suggested that AQP localization to the leading edge of migrating cells facilitates uptake of water, thereby causing a small swelling that facilitates forward movement by generating increased space for actin filaments [100,101,102,103] (for review [104,105]). Several studies show that AQP3 may also contribute to cancer progression by increasing the motility and invasiveness of cancer cells. Increased AQP3 expression correlated with higher histopathological grade and increased spreading to lymph nodes in patients with invasive breast ductal carcinoma [83]. In addition, AQP3 knockdown abrogated metastasis to lungs from mammary orthotopic xenographs in vivo [26], supporting the role of AQP3 in cancer metastasis. In cell cultures, AQP3 expression was associated with increased migration and invasion of T47D [83], MDA-MB-231, and DU4475 [26] breast cancer cells, while knockdown of AQP3 significantly reduced migration compared to control cells. Moreover, AQP3-facilitated transport of H_2_O_2_ into cells was necessary to regulate protein kinase B (Akt) phosphorylation and subsequent directional cell migration of chemokine (C-X-C motif) ligand 2 (CXCL2)-dependent breast cancer cells in vitro [26]. Together, these results suggest AQP3 as a driver of motility. Future experiments are necessary to clarify the contribution of AQP3-mediated transport of H_2_O and H_2_O_2_ in cell movement, and if AQP3 accelerates movement through stimulation of intracellular signaling pathways (Figure 2).

### 2.4. Cancer Cell Invasion

An important aspect of cancer metastasis is the ability to degrade the extracellular matrix (ECM) and invade the neighboring tissue. Matrix metalloproteases (MMPs) break down ECM proteins and facilitate migration and invasion of cancer cells. Interestingly, AQP3 overexpression positively regulated the levels of membrane type 1-matrix metalloprotease (MT1-MMP), MMP2 and MMP9, while AQP3 knockdown resulted in reduced levels in SCG7901 human gastric cancer cells [94] (Figure 2). Moreover, AQP3 silencing reduced invasiveness of DU-145 and PC-3 prostate cancer cells which was paralleled with decreased mRNA levels and hence activity of MMP3 [95]. Inhibition of extracellular signal-regulated kinase (Erk) 1/2 activation also blunted these processes [95], but so far, the mechanisms connecting AQP3 to the Erk 1/2 pathway remains to be characterized.

### 2.5. Contribution to EMT

Evidence implicates that overexpression of AQP3 may aggravate EMT of cancer cells. During EMT, epithelial cells convert to a more mesenchymal morphology. Cell–cell adhesions are weakened and apical-basal polarity changes to a more front-rear polarity. This is mediated by several transcriptional changes including altered expression profile of cell adhesion molecules and disruption of polarity complexes as well as cytoskeleton reorganization, which increases migration (for reviews [106,107,108]). Several studies of in vitro cell culture and animal tumor models have illustrated the necessary role of growth factor-induced EMT in metastasis of multiple cancers including non-small-cell lung cancer [109], breast cancer [110], liver cancer [111], colon cancer [112], head and neck squamous cell carcinoma [113], ovarian cancer [114], cervical cancer [115], bladder cancer [116], and esophageal cancer [117].

Involvement of AQP3 in increased cellular motility and invasiveness support a role in EMT. Moreover, a few studies have directly implicated AQP3 in EMT progression in cancer cells. In T47D breast cancer cells, overexpression of AQP3 caused decreased protein levels of E-cadherin, while Snail expression was increased [83]. In contrast, total protein levels of β-catenin were unchanged [83]. Similarly, in a study using the gastric cancer cell lines SGC7901 and MGC803, overexpression of AQP3 correlated with down-regulation of E-cadherin expression and up-regulation of vimentin and fibronectin expression [84]. In addition, T-cell factor (TCF) and lymphocyte enhancer factor (LEF), transcription factors known to be involved in EMT (for review [106,107]), were also affected by AQP3. AQP3 knockdown decreased both TCF and LEF expression levels in SGC7901 and MGC803 gastric cancer cells [98]. Thus, the mechanisms underlying regulation of EMT by AQP3 overexpression is an important future research topic to unravel the role of AQP3 in cancer.

## 3. Conclusions and Perspectives

The reports discussed here demonstrate increased AQP3 expression in many cancers, and that AQP3 overexpression contributes to cancer development. In cell culture studies, increased AQP3 expression enhances cancer-associated cellular mechanisms such as proliferation, migration and invasion, and aggravates EMT progression of cancer cells. Knock-out and knockdown experiments have shown that these effects are dependent on AQP3. Many aspects of AQP3 in cancer still need to be clarified.

Upstream AQP3 regulators have been identified. Expression of AQP3 is stimulated by hormones that are commonly increased in cancer, including EGF and estrogen. Estrogen receptor acted as a transcriptional regulator of AQP3. Similarly, during keratinocyte differentiation, the AQP3 gene has been reported as a transcriptional target of Notch signaling [118]. Future studies may further elucidate the role of upstream regulation of AQP3 in specific cancer types.

It is also important to identify if solute/water transport by AQP3 alone can explain the effect of AQP3 in cancer development, or if AQP3 can act through other mechanisms. As discussed, AQP-mediated solute and water transport may contribute to cancer progression. Since cancer cells have increased levels of H_2_O_2_ [119], AQP3-meditated H_2_O_2_ transport may play a role in breast cancer progression [26]. Further research is required to determine the importance of the H_2_O_2_ transport function of AQP3 in other cancers and the downstream signaling mechanisms. Moreover, it is of interest to identify any other potential solutes that may be transported by AQP3 and act as contributors to cancer development. Other AQPs (for reviews [78,80,81,120]) have been shown to stimulate intracellular signaling in cancer cells. AQP3 may be an upstream regulator of intracellular signaling cascades, including Akt and Erk 1/2 signaling. Unlike AQP2 or AQP5, phosphorylation or ubiquitination sites that could participate in signaling have not been identified for AQP3. It is of importance to identify if AQP3 may participate in intracellular signaling via the transport capacity or other pathways. These studies will provide further insight and advance our understanding of the molecular mechanisms behind the contribution of AQP3 to cancer metastasis. Since there are currently no efficient drugs directly targeting AQP3 expression levels, it is crucial to identify downstream signaling pathways that are stimulated by AQP3 and can be used as drug targets.
